# Editorial: Executive Function and Education

**DOI:** 10.3389/fpsyg.2018.01357

**Published:** 2018-08-03

**Authors:** Mariëtte Huizinga, Dieter Baeyens, Jacob A. Burack

**Affiliations:** ^1^Department of Educational and Family Studies, LEARN! Research Institute, Vrije Universiteit Amsterdam, Amsterdam, Netherlands; ^2^Faculty of Psychology and Educational Sciences, KU Leuven, Leuven, Belgium; ^3^Department of Educational and Counselling Psychology, McGill University, Montreal, QC, Canada

**Keywords:** executive function, education, academic achievement, intervention, development, parents, family, teachers

Executive function is an umbrella term for various cognitive processes that are central to goal-directed behavior, thoughts, and emotions. These processes are especially important in novel or demanding situations, which require a rapid and flexible adjustment of behavior to the changing demands of the environment. The development of executive function relies on the maturation of associated brain regions as well as on stimulation in the child's social contexts, especially the home and school. Over the past decade, the term executive function has become a buzzword in the field of education as both researchers and educators underscore the importance of skills like goal setting, planning, and organizing in academic success. Accordingly, in initiating this Research Topic/eBook our goal was to provide a forum for state-of-the-art theoretical and empirical work on this both facilitates communication among researchers from diverse fields and provides a theoretically sound source of information for educators. The contributors to this volume, who hail from several different countries in Europe and North America, have certainly accomplished this goal in their nuanced and cutting-edge depictions of the complex links among various executive function components and educational success.

In trying to present a coherent presentation of the many excellent contributions, we conceptually divided the papers in this Research Topic/eBook into the three broad sections of (1) executive function as predictor for academic outcomes, (2) teacher, parent, and family factors in the relationship between executive function and academic outcomes, and (3) interventions and their impact on executive function and academic performance.

## Executive function as predictor for academic outcomes

The first section of this Research Topic/eBook is focused on executive function as predictors of academic outcomes. All five papers are empirical reports on the extent to which executive function very early in the child's life predict educational success later in childhood (Daucourt et al.; Dekker et al.; Mulder et al.; Ribner et al.; Von Suchodoletz et al.). In a longitudinal study among 552 children in the Netherlands, Mulder et al. find that executive function abilities at age 2 years are significant and relatively strong predictors of both emergent mathematics and literacy tasks at age 5 years, after controlling for receptive vocabulary, parental education, and home language. In a longitudinal study of a sample of 1,292 children between the ages of ~10.5 and 12.5 years from low-income families in the United States, Ribner et al. highlight that, in addition to being a unique predictor of success in both 5th grade math and reading, high levels of early executive function can help to compensate for low levels of academic ability in Pre-K. In a longitudinal study of a hybrid model of reading disability (a composite consisting of four symptoms, including low word reading achievement, unexpected low word reading achievement, poorer reading comprehension compared to listening comprehension, and dual-discrepancy response-to-intervention), Daucourt et al. find that three of the components of executive function—inhibition, updating working memory, and shifting—are similarly predictive of subsequent reading disability among a group of 420 children between the ages of almost 5–10.5 years in the United States. Similarly, in study in The Netherlands, Dekker et al. find that teacher and child measures of working memory and shifting are significantly associated with math and spelling outcome among first and second graders (range 6.25–8.5 years old; *N* = 84). In considering individual differences among a group of 69 first, 121 third, and 85 eighth grade students (mean ages 7.2, 8.5, and 14 years, respectively) in Germany, Von Suchodoletz et al. find that attention shifting is related to spelling outcomes for all three age groups, but that this relationship is further specified by sex differences among the first and eight graders.

## Teacher, parent, and family factors in the relationship between executive function and academic outcomes

The four articles in the second section highlight the notion that the study of development of executive function in relation to academic outcomes cannot be confined solely to the study of the child, but must be broadened to include the impact of the essential persons and contexts in the child's life, including teachers, parents, and family situation. Two papers are focused on the role of parental and teacher support on the impact of executive function on school performance (Devine et al.; Vandenbroucke et al.) and the other two on the impact of family risk factors on the relationship between executive function and educational success (Berthelsen et al.; Welsh et al.). In a longitudinal study of 117 parent-child dyads in the United Kingdom, with children between the ages of 3 and 4 at baseline, Devine et al. find that three aspects of parental behavior—parental scaffolding, negative parent-child interactions, and the provision of informal learning opportunities—are unrelated to each other and all show unique contributions to children's early academic ability as executive function mediates the relations between parental scaffolding and negative parent-child interaction and children's early academic ability. In contrast, parental provision of opportunities for learning in the home environment was directly related to children's academic abilities. In a Belgian study of the role of parent and teacher emotional support in promoting working memory performance by buffering the negative effect of social stress in 170 children in grades 1 and 2 (mean age = 7.6 years), Vandenbroucke et al. find that parents and teachers can have a substantial influence on children's working memory performance by offering adequate emotional support, confirming the idea that cognitive processes, such as working memory, do not merely depend on maturation but can also be supported or hindered by environmental factors. Drawing on wave 1 (4–5 years old) and wave 6 (14–15 years old) from the *Growing up in Australia: The Longitudinal Study of Australian Children* (*N* = 4,983), Berthelsen et al. find that higher child behavior risk, lower socio-economic position, and child behavior risk are associated with poorer executive function in adolescence. While the effects of the early ecological risk on the development of executive function are relatively small, they operate through children's early self-regulatory behaviors of attentional regulation and approaches to learning, at the beginning of the school years. In an initial study in the United States on the deleterious outcomes of a self-reported history of child-maltreatment (including emotional and physical abuse and neglect, and sexual abuse) in relation to college academic outcomes in terms of GPA and self-reported adjustment among 64 students, Welsh et al. find that relatively “hot” executive function serve as a link among child maltreatment experiences and college achievement and adaptation.

## Interventions and their impact on executive function and academic outcomes

The articles in the final section are focused on interventions and their impact on executive function and academic performance (Kamkar and Morton; Solomon et al.; Stein et al.; Zelazo et al.). In a study of 101 children in German kindergarten randomly assigned either to a coordinative intervention or to a control condition, Stein et al. find no effect of the intervention on executive function of children in a kindergarten setting. In a pre-test, post-test, follow-up randomized-control trial in the United States of 218 preschool children (mean age = 4.75 years) from schools serving low-income families randomly assigned to Mindfulness + Reflection training; Literacy training; or Business as Usual (BAU) options delivered by trained teachers in 30 small-group sessions over 6 weeks, Zelazo et al. find that that executive function improved in all groups, but the Mindfulness + Reflection group significantly outperformed the BAU group, which did not differ from the literacy group, at follow-up. In Canada, in a cluster-randomized controlled trial of 260 3- and 4 year-olds assigned to either the Tools of the Mind preschool curriculum designed to target self-regulation through imaginative play and self-regulatory language or Playing to Learn (another play-based program that does not target self-regulation specifically), Solomon et al. find no effect of curriculum on any of the outcome measures although children with high levels of hyperactivity/inattention who received Tools instruction showed greater improvement in self-regulation. In a conceptual paper based on empirical evidence, Kamkar and Morton propose the CanDiD framework in which they highlight that dynamic and contextual influences on EF must be considered in relation to development and individual differences, and that these factors are relevant to remedial interventions and curriculum design.

## Conclusions and future directions

This collection of papers highlights that executive function is pivotal for academic achievement. The link is already apparent at preschool age when executive function predicts emergent mathematics and literacy skills (Mulder et al.). In addition, later on in development, working memory, inhibition, and cognitive flexibility are all predictors of (disabilities in) mathematics, reading and spelling in primary and secondary education (Daucourt et al.; Dekker et al.; Ribner et al.; Von Suchodoletz et al.). The predictive value of executive function for academic achievement seems to be robust for controlling measures of socio-economic status, home language, receptive vocabulary, etc., as well as for national differences in schooling systems.

Although brain maturation is important for the development of executive function especially in periods of rapid growth, this development is highly sensitive to influences from environmental factors (Anderson et al., 2008). Yet, researchers have only recently began to focus on the impact of children's social environment on EF development (Hughes, 2011). The importance of both distal and proximal parent and family factors (e.g., parental scaffolding, negative parent-child interactions) as well as characteristics of the teacher-child interactions (e.g., emotional support) for executive function development and in turn academic achievement are stressed in several papers in this collection (Berthelsen et al.; Devine et al.; Vandenbroucke et al.; Welsh et al.). In line with the CanDiD framework (Kamkar and Morton), the findings from these studies suggest that context factors should be taken into account in remedial interventions and curriculum design.

As indicated by the current collection of intervention studies, executive function training programs (1) seem to evolve into broader intervention programs, which are generally implemented in the specific context where the executive function should be applied for the actions of interest (e.g., reading, spelling, mathematics) and (2) should be individually tailored to the needs of the particular child in order to deal with inter-individual differences in executive function performance and development (Solomon et al.; Stein et al.; Zelazo et al.). By doing so, the central role of executive function in educational practice can be stimulated and optimized.

We thank the contributors for their thoughtful and provocative contributions to this Research Topic/eBook and hope that this collection will both add to the current literature and serve as foundation for future empirical and applied work to better the academic outcomes of children worldwide.

## Author contributions

All authors listed have made a substantial, direct and intellectual contribution to the work, and approved it for publication.

### Conflict of interest statement

The authors declare that the research was conducted in the absence of any commercial or financial relationships that could be construed as a potential conflict of interest.
